# Cost-effectiveness model of trastuzumab deruxtecan as second-line treatment in HER2-positive unresectable and/or metastatic breast cancer in Finland

**DOI:** 10.1007/s10198-023-01617-3

**Published:** 2023-07-24

**Authors:** Jeroen H. J. Paulissen, Ahmed H. Seddik, Kyle J. Dunton, Christopher J. Livings, Marinus van Hulst, Maarten J. Postma, Lisa A. de Jong, Roel D. Freriks

**Affiliations:** 1 Department of Health Sciences, University Medical Center Groningen, Groningen, The Netherlands; 2 Asc Academics, Groningen, The Netherlands; 3 Daiichi Sankyo Europe GmbH, Munich, Germany; 4 Daiichi Sankyo UK Ltd, Uxbridge, UK; 5 AstraZeneca Plc., Cambridge, UK; 6 Department of Clinical Pharmacy and Toxicology, Martini Hospital, Groningen, The Netherlands; 7 Department of Economics, Econometrics and Finance, Faculty of Economics & Business, University of Groningen, Groningen, The Netherlands

**Keywords:** Cost-effectiveness, Breast cancer, Metastatic, HER2-positive, Trastuzumab deruxtecan, D61

## Abstract

**Objectives:**

Trastuzumab deruxtecan (T-DXd) was recently recommended by the Committee for Medicinal Products for Human Use as a treatment for adult patients with unresectable or metastatic HER2-positive breast cancer, who had received a prior anti-HER2-based regimen. In our study, we evaluated the cost-effectiveness of T-DXd compared with ado-trastuzumab emtansine (T-DM1) for this indication in Finland.

**Methods:**

A three-state partitioned survival analysis model was developed with a payer’s perspective. Time to event data from the DESTINY-Breast03 (DB-03) trial were extrapolated over a lifetime horizon either directly—for progression-free survival and time to treatment discontinuation—or using an alternative approach utilizing long-term T-DM1 survival data and DB-03 data—for overall survival. Discount rates of 3% were applied for costs and effects. Inputs were sourced from the Medicinal Products Database from Kela, Helsinki University Hospital service price list, Finnish Medicines Agency assessments, clinical experts, and DB-03. Sensitivity analyses were performed to characterize and demonstrate parameter uncertainties in the model.

**Results:**

Total quality-adjusted life years (QALYs) and life years (LYs) gained for T-DXd compared with T-DM1 were 1.93 and 2.56, respectively. Incremental costs for T-DXd compared with T-DM1 were €106,800, resulting in an ICER of €55,360 per QALY gained and an ICER of €41,775 per LY gained. One-way sensitivity analysis showed the hazard ratio of T-DXd vs T-DM1 for OS was the most influential parameter. The probabilistic sensitivity analysis showed similar results to the base case.

**Conclusions:**

T-DXd is cost-effective based on surrogate WTP thresholds of €72,000 and €139,000 per QALY.

**Supplementary Information:**

The online version contains supplementary material available at 10.1007/s10198-023-01617-3.

## Introduction

Breast cancer was the cause of 968 deaths in Finland in 2020 and, with 4885 new cases that year, it was also the most common newly diagnosed cancer [1]. Patients with unresectable and/or metastatic breast cancer (uBC and/or mBC) experience significantly impacted quality of life [2], and disease management is associated with high costs [3]. Though mBC is treatable, it remains incurable, and breast cancer subtype is a strong predictor of survival [4]. A subtype of interest for targeted treatments is human epidermal growth factor receptor 2 overexpressing (HER2-positive) disease, which accounts for approximately 15–20% of breast cancers [5].

In the past two decades, treatment advances and the rise of monoclonal antibodies have improved survival outcomes for HER2-positive advanced breast cancer patients, and targeted therapies are approved for multiple lines in the treatment pathway for HER2-positive mBC [6]. In the first line, the European Society for Medical Oncology (ESMO) clinical practices guidelines for advanced breast cancer recommend the combination of trastuzumab–pertuzumab–taxane as standard for HER2-positive mBC [7]. In the second line, ado-trastuzumab emtansine (T-DM1) had been the gold standard for this indication since 2012 based on the EMILIA trial results, and until recently, there had been no advances in second-line treatment since its introduction [4, 7].

Trastuzumab deruxtecan (T-DXd) is an HER2-directed antibody–drug conjugate that was recently recommended by the Committee for Medicinal Products for Human Use for approval as indicated for adult patients with HER2-positive uBC and/or mBC who had received a prior anti-HER2-based regimen [8]. Approval was recommended based on results from the phase 3 DESTINY-Breast03 (DB-03) trial, which compared T-DXd with T-DM1 in patients previously treated with a taxane and trastuzumab. T-DXd demonstrated improved progression-free survival (PFS, hazard ratio [HR] 0.28; 95% CI 0.22 to 0.37; *p* < 0.001) and overall survival (OS, HR 0.55; 95% CI 0.36 to 0.86; *p* = 0.007, prespecified significance boundary not reached) compared with T-DM1 [9]. The ESMO guidelines now recommend T-DXd as the preferred second-line treatment for HER2-positive uBC and/or mBC [7].

The aim of this study was to evaluate the cost-effectiveness of T-DXd compared with T-DM1 as a second-line treatment for adult patients with HER2-positive uBC and/or mBC previously treated with trastuzumab and a taxane in Finland.

## Methods

A partitioned survival analysis (PartSA) model, widely used to assess cost-effectiveness in oncology treatments, was developed in Microsoft Excel (2016) to evaluate the cost-effectiveness of T-DXd compared with T-DM1 as a second-line treatment for adult patients with HER2-positive uBC and/or mBC previously treated with trastuzumab and a taxane in Finland. The model compared T-DXd with T-DM1, and model structure and inputs were determined using clinical trial data (DB-03; data cutoff date: May 21, 2021; median duration of follow-up: 16.2 months [0–32.7] with T-DXd and 15.3 months [0–31.3] with T-DM1), literature reviews, and consultation with clinical and health economic experts. The analysis was conducted from a payer’s perspective, aligned with previous Fimea assessments [10, 11]. Outcomes were the incremental cost-effectiveness ratio (ICER) calculated as cost per quality-adjusted life year (QALY) gained and the ICER calculated as cost per life year (LY) gained. A discount rate for costs and effects of 3% per annum was applied [12]. The cycle length was 3 weeks, aligned with the dosing schedule from DB-03. This cycle length was short enough to capture clinically and economically meaningful events between cycles. Half-cycle correction was applied. A lifetime horizon of 41 years was used in the base case analysis to capture all essential costs and health effects [12]. Additionally, several analyses were performed to determine the model sensitivity to parameter uncertainty, including a one-way sensitivity analysis (OWSA) and a probabilistic sensitivity analysis (PSA), and scenario analyses were performed to assess the robustness of the model.

The study followed the Professional Society for Health Economics and Outcomes Research (ISPOR) Consolidated Health Economic Evaluation Reporting Standards (CHEERS) checklist and adhered to good reporting practice for health economic evaluations [13].

### Patient characteristics

The modelled cohort comprised patients aged 18 years or older with HER2-positive uBC and/or mBC previously treated with trastuzumab and a taxane, based on the patient population from the DB-03 trial [9]. The mean age of the model cohort was 54.4 years, the proportion of female patients was 99.6%, and the average weight and body surface area were 62.40 kg and 1.65m^2^, respectively (Table 1), based on the unpublished DB-03 clinical study report. These characteristics were used to calculate age- and sex-dependent inputs (such as general population mortality) and drug dosing requirements.

**Table 1:** Summary of model inputs for patient characteristics and medical, resource use, and adverse event costs

Parameter	Model input			Reference
**Patient characteristics**				
Mean age	54.4 years			DB-03 clinical study report
Percent female	99.6%			
Average weight	62.40 kg			
Average body surface area	1.65 m^2^			
**Costs**				
Drug	Costs (per vial)		Dose (every 3 weeks)	
T-DXd 100 mg	€1660.00		5.4 mg/kg	DB-03 clinical study report, Kela [14]
T-DM1 100 mg	€1817.45		3.6 mg/kg	
T-DM1 160 mg	€2907.92		3.6 mg/kg	
**Administration costs**				
Oral	€0			Assumption
Parenteral	€160^a^			HUH [15]
**Health state utilities**				
	T-DXd		T-DM1	
Progression free	0.8181		0.8009	DB-03 clinical study report
Progressed	0.5403		0.5403	DB-03 clinical study report, Lloyd et al. [16]
**Resource use and costs**				
Resource	Cost		Number per cycle	
Specialist physician/oncologist^**b**^	€86.86^c^		0.27	Mäklin et al., Fimea T-DXd [10, 17]
Blood tests^**b**^	€21.24^d^		1.02	[10, 17]
ECHO/MUGA-scanning, cardiological examination^**b**^	€269.99^e^		0.09	[10, 17]
CT scanning^**b**^	€161.16^f^		0.27	[10, 17]
Nurse visit^**b**^	€36.63^c^		0.45	[10, 17]
End of life				
Last 60 days of life	€9148.17		20%	Haltia et al. [18]
**Adverse event costs (per event)**				
Adverse event	Costs			
Neutrophil count decrease	€258.48			Mäklin et al. [17]
Anaemia	€1189.86			Mäklin et al., Fimea Nivolumab [11, 17]
White blood cell decrease	€258.48			[17]
Platelet count decrease	€258.48			[17]
Nausea	€238.60			[17]
Increased AST	€ 238.60			[17]
ILD	€4112.70			[17]
LVEF decrease	€3189.70			[17]

*AST* aspartate aminotransferase, *CT* computerized tomography, *DB*-*03* DESTINY-Breast03, *ECHO* echocardiogram, *HUH* Helsinki University Hospital, *ILD* interstitial lung disease, *LVEF* left ventricular ejection fraction, *MUGA* multigated acquisition, *T*-*DM1* ado-trastuzumab emtansine, *T*-*DXd* trastuzumab deruxtecan

^a^V1182010/V1182011/V1182012

^b^Used in both progression-free and post-progression health states

^c^Outpatient care

^d^Codes combined: 2474, 1552, 2203, 4520, 4594, 2516/6024, 4586, 1999, 1046/4587, 1128/4591, 1026/1024

^e^NA3BG

^f^NA3AD

### Model structure

**Fig. 1:**
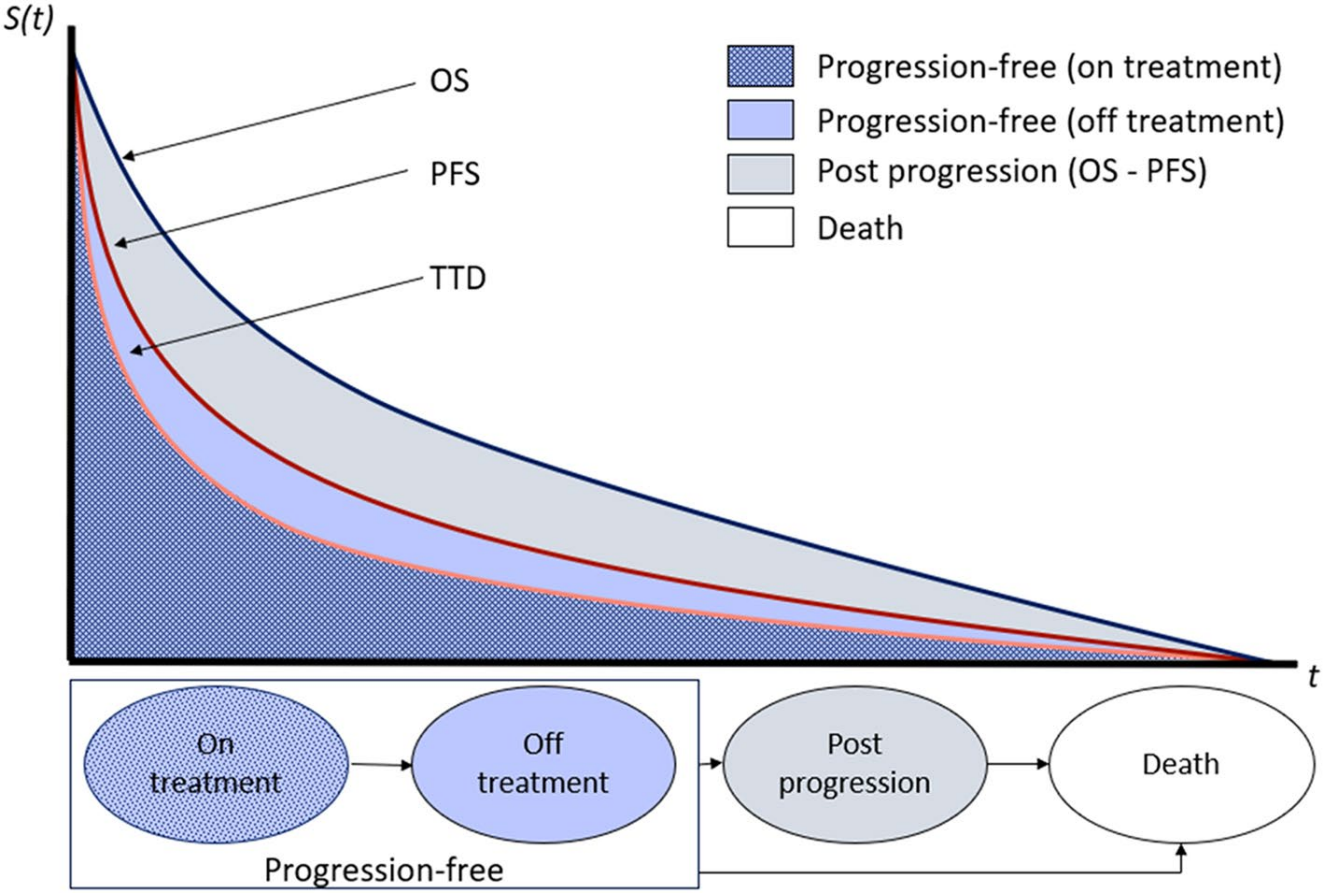
Structure of the PartSA model demonstrating the health state membership across time for the progression-free (on treatment and off treatment), post-progression, and death health states. *OS* overall survival, *PartSA* partitioned survival analysis, *PFS* progression-free survival, *S*(*t*) survival over time, *t* time, *TTD* time to treatment discontinuation

A three-state PartSA model was developed consisting of three health states: progression free, post-progression, and death (Fig. 1). Health states were mutually exclusive. Patients entered the model in the progression-free state and received either T-DXd or T-DM1. Patients in this state could remain progression free, their disease could progress, or they could die. Patients could remain progression free while receiving T-DXd or T-DM1 (on treatment) or patients could remain progression free without receiving treatment (off treatment). Off-treatment patients were not able to return to treatment at a later time point. Patients in the post-progression state could not return to the progression-free state, but they could remain alive in the post-progression state, or they could die.

State membership was determined from the PFS and OS curves reported in the DB-03 trial, as shown in Fig. 1. PFS was used to determine the proportion of patients in the progression-free health state over time. OS was used to inform membership in the death state. For the post-progression state, state membership was derived from the difference between the OS and PFS curve at each time point. Time to treatment discontinuation (TTD) data from DB-03 was used to distinguish between patients in the progression-free state who are on treatment and those who are off treatment.

### Clinical parameters

Standard parametric models—exponential, Weibull, Gompertz, log-logistic, log-normal, generalized gamma, and gamma—were fitted to the TTD and PFS data from DB-03 using their distribution parameters (Table 1—Supplementary Information 1) [12]. The best fit was selected based on goodness-of-fit statistics, visual inspection, and clinical plausibility of the long-term extrapolations. For OS, an alternative approach was used, described in detail below.

#### Time to treatment discontinuation and progression-free survival

**Fig. 2:**
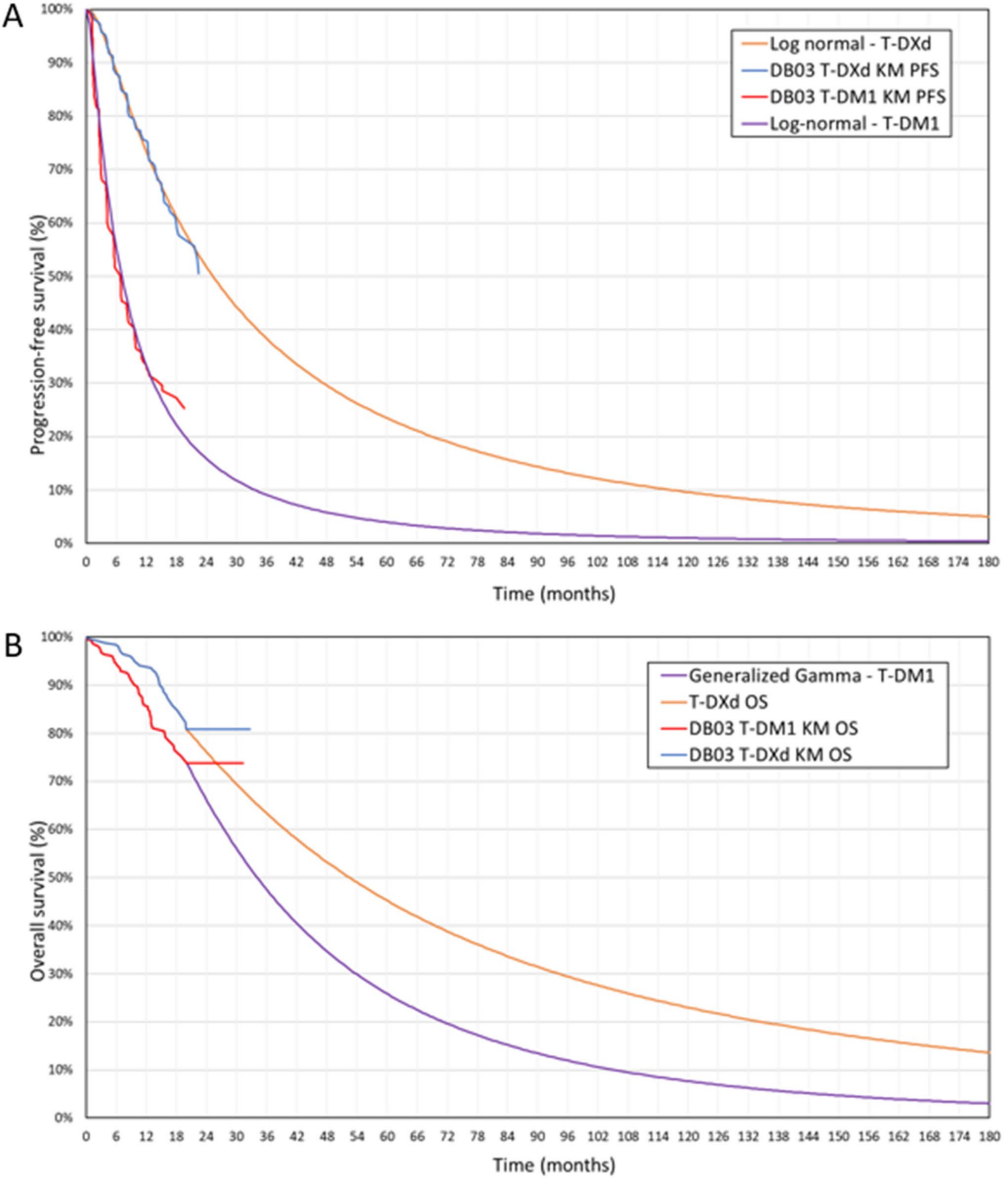
Kaplan–Meier survival curves and resulting survival curves used in the model for PFS (**A**) and OS (**B**). Kaplan–Meier curves were constructed based on the unpublished DB-03 clinical study report. Log-normal parametric distributions were fitted to the Kaplan–Meier curve for PFS of T-DXd and T-DM1. Generalized gamma parametric distribution was fitted to the digitized T-DM1 Kaplan–Meier curve for OS from EMILIA [1] and attached to the Kaplan–Meier survival curve to create T-DM1 OS estimates. The resulting OS curve for T-DM1, Kaplan–Meier survival curve for T-DXd, and HR from DB-03 were used to create T-DXd OS estimates. *DB-03* DESTINY-Breast03, *KM* Kaplan–Meier, *OS* overall survival, *PFS* progression-free survival, *T*-*DM1* ado-trastuzumab emtansine, and *T*-*DXd* trastuzumab deruxtecan

In the base case, TTD data from DB-03 were directly extrapolated to estimate TTD for patients in the T-DM1 and T-DXd arms, and the log-normal distribution was used for both arms. PFS data from DB-03 were also directly extrapolated to estimate PFS for patients in the T-DM1 and T-DXd arms in the base case. The log-normal was the best fit for T-DXd, while the generalized gamma provided the best fit for T-DM1; however, the Pharmaceutical Pricing Board guidelines suggest fitting separate parametric models of the same type to both arms [12]. Furthermore, the log-normal distribution resulted in the closest median PFS to the median PFS observed in DB-03 for T-DXd (investigator assessed) and T-DM1 (assessed by BICR)—25.1 and 6.8 months, respectively (Tables 3 and 5—Supplementary Information 1), based on the unpublished DB-03 clinical study report. Thus, log-normal distributions were used for both arms (Table 2 and 4—Supplementary Information 1). The resulting PFS curves for T-DXd and T-DM1 are shown in Fig. 2.

#### Overall survival

OS event rates from DB-03 were not high enough to inform long-term direct extrapolations. For this reason, an alternative approach adapted from similar published methods was used in the base case [19, 20]. This approach generates a curve using data from two sources: DB-03 OS KM data was used for both T-DXd and T-DM1 until the time point at which the last death was observed (20 months). After 20 months, the curves for both T-DXd and T-DM1 were generated using long-term T-DM1 OS data from the EMILIA clinical trial and the HR for T-DXd versus T-DM1 from the unpublished DB-03 clinical study report (0.56, 95% CI 0.36 to 0.86) [21]. The EMILIA trial compared T-DM1 to lapatinib plus capecitabine in patients with HER2-positive advanced breast cancer who had previously been treated with trastuzumab and a taxane, with a follow-up of 20 months [21]. The EMILIA data was validated through multiple methods (provided in Supplementary Information 2) and deemed to reflect anticipated long-term outcomes for the DB-03 T-DM1 arm.

The approach comprised the following steps:

1. Standard parametric models were fitted to the long-term EMILIA OS for T-DM1 and assessed on goodness-of-fit statistics, visual inspection, and clinical plausibility of the extrapolations. Since standard parametric models were not fitted to DB-03 T-DM1 data directly, an additional analysis of clinical plausibility was performed (Supplementary Information 2). The generalized gamma distribution was selected as the best fit and used to generate the extrapolated tail of the curve.
2. Two hazard rate functions were calculated: one based on the T-DM1 curve resulting from the previous step (after 20 months), and one based on the DB-03 T-DM1 data (until 20 months only).
3. These two hazard rate functions were combined into one hazard rate function by assigning the hazard rates calculated from the DB-03 T-DM1 data to each time point until 20 months and assigning the hazard rates from the generalized gamma fit to EMILIA T-DM1 to each time point from 20 months onwards.
4. The T-DM1 OS curve for the model was derived from this combined hazard rate function.
5. The T-DXd OS curve was constructed using the KM data from DB-03, the derived T-DM1 OS curve, and the HR from DB-03. Until 20 months, the T-DXd OS curve was based on the KM data from DB-03. After 20 months, the HR from DB-03 was applied to the T-DM1 OS curve to calculate the T-DXd OS curve. Combining these together creates the final T-DXd OS curve used in the model. The resulting OS curves for T-DXd and T-DM1 are shown in Fig. 2.

### Adverse events

The base case analysis included the costs associated with adverse events (AEs) with a severity of Grade 3 or higher when 5% or more patients in one of the treatment arms experienced AE. Additionally, costs associated with interstitial lung disease and decrease in left ventricular ejection fraction were included in the model regardless of incidence or severity, as these were considered AEs of special interest in DB-03. Incidence of AEs for T-DXd and T-DM1 were sourced from the unpublished DB-03 clinical study report. AE costs were only incurred in the first cycle of the model, concurrent with treatment initiation.

### Health state utilities

Treatment-specific progression-free health state utilities (Table 1) were sourced from the DB-03 trial. It was assumed these captured disutility. Post-progression health state utilities (Table 1) were derived from the algorithm developed by Lloyd et al. using overall baseline characteristics from the unpublished DB-03 clinical study report [16] (Eq. 1—Supplementary Information 1).

### Costs and resource use

Costs included in the model were drug and administration costs, costs of disease management, end-of-life costs, and treatment costs for AEs (summarized in Table 1). Drug costs were sourced from the Medicinal Products Database from Kela [14]. Administration costs were sourced from the Helsinki University Hospital service price list [15]. Type and frequency of resource use were sourced from previous technology assessments by the Finnish Medicines Agency (Fimea) and validated by Finnish clinical experts [10]. Resource costs and AE-related costs were sourced from the Finnish Institute for Health and Welfare or previous Fimea assessments and have been adjusted to 2021 using consumer price index from the Statistics Finland [11, 17, 22].

Subsequent treatment costs—consisting of drug and administration costs—were sourced from previous Fimea assessments [10, 23]. The proportion of patients receiving a specific treatment option after T-DXd or T-DM1 was based on Finnish clinical expert opinion (Table 7—Supplementary Information 1). The proportion of patients receiving any treatment option as subsequent treatment was sourced from the unpublished DB-03 clinical study report—i.e. 60.7% and 76.1% for patients in the T-DXd arm and T-DM1 arm, respectively. It was assumed that patients received subsequent treatment until death.

### Relative dose intensity

A relative dose intensity (RDI) was applied to the T-DXd and T-DM1 dose. The RDI was taken from the unpublished DB-03 clinical study report.

### Sensitivity and scenario analyses

#### One-way sensitivity analysis (OWSA)

An OWSA was conducted to demonstrate the impact of parameter uncertainty by varying parameters individually within their 95% confidence interval or by plus or minus 20% of the base case value. Parameters with set values (e.g. drug costs) were not varied as they do not bring uncertainty to the model.

#### Probabilistic sensitivity analysis (PSA)

A PSA was conducted to comprehensively characterize parameter uncertainty by varying all input parameters for 1000 iterations. The cost parameters were sampled from gamma distributions, and the HR from DB-03 of T-DXd versus T-DM1 for OS was sampled from a log-normal distribution. Other inputs, such as utility values, incidence of AEs, incidence of AE-related hospitalizations, proportions of patients, and RDI were sampled from beta distribution. To account for correlation between parameters in survival distributions, a Cholesky decomposition algorithm was used [24].

#### Scenario analysis

The model also incorporated scenario analyses exploring structural model uncertainty by changing the model time horizon, discount rates, vial sharing, no half-cycle correction, background mortality, the proportion of patients receiving subsequent therapy, the health state utility source, and provides a scenario in which the DB-03 OS data was directly extrapolated using the distribution with the best statistical fit (i.e. log-logistic).

## Results

### Base case results

Over a lifetime horizon, the deterministic results show the total QALYs and LYs gained for T-DXd compared to T-DM1 were 1.93 and 2.56, respectively. Incremental costs for T-DXd compared to T-DM1 were €106,800, resulting in an ICER of €55,360 per QALY gained and an ICER of €41,775 per LY gained.

### Sensitivity and scenario analyses

#### One-way sensitivity analysis

**Fig. 3:**
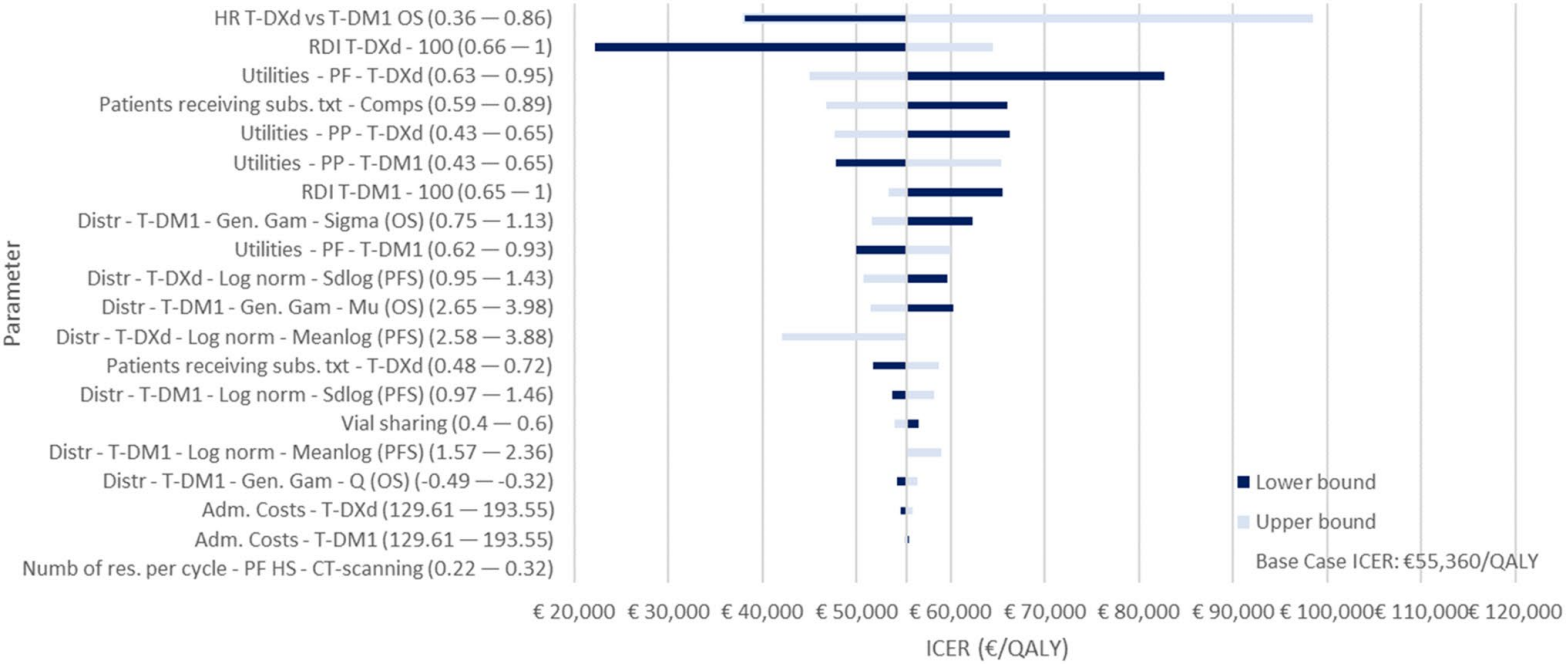
Tornado diagram of the ICER of T-DXd versus T-DM1. *Adm* administration, *CT* computerized tomography, *Distr* distribution, *gen*. *gam* generalized gamma, *HR* hazard ratio, *ICER* incremental cost-effectiveness ratio, *Log norm* log-normal, *OS* overall survival, *PF* progression free, *PFS* progression-free survival, *PP* post-progression, *Prop* proportion, *RDI* relative dose intensity, *Res* resources, *T*-*DXd* trastuzumab deruxtecan, *T-DM1* ado-trastuzumab emtansine, *Txt* treatment, *Subs*. subsequent treatment

The 20 most influential parameters in the OWSA are presented in Fig. 3. The results show that parameter uncertainty has the greatest impact on the ICER for the following: the HR of T-DXd vs T-DM1 for OS; the RDI used for T-DXd; the progression-free utility value for T-DXd; the patients in the T-DM1 arm receiving any subsequent treatment; and the post-progression utility value for T-DXd.

#### Probabilistic sensitivity analysis

The mean probabilistic results (using the average costs and QALYs of all iterations) generated by varying the base case settings randomly within their respective boundaries produced a mean ICER of €56,084 per QALY gained for T-DXd compared with T-DM1 (Table 8—Supplementary Information 1). The mean ICER resulting from the PSA is close to the base case ICER of €55,360 per QALY, which demonstrates the robustness of the model and the base case.

**Fig. 4:**
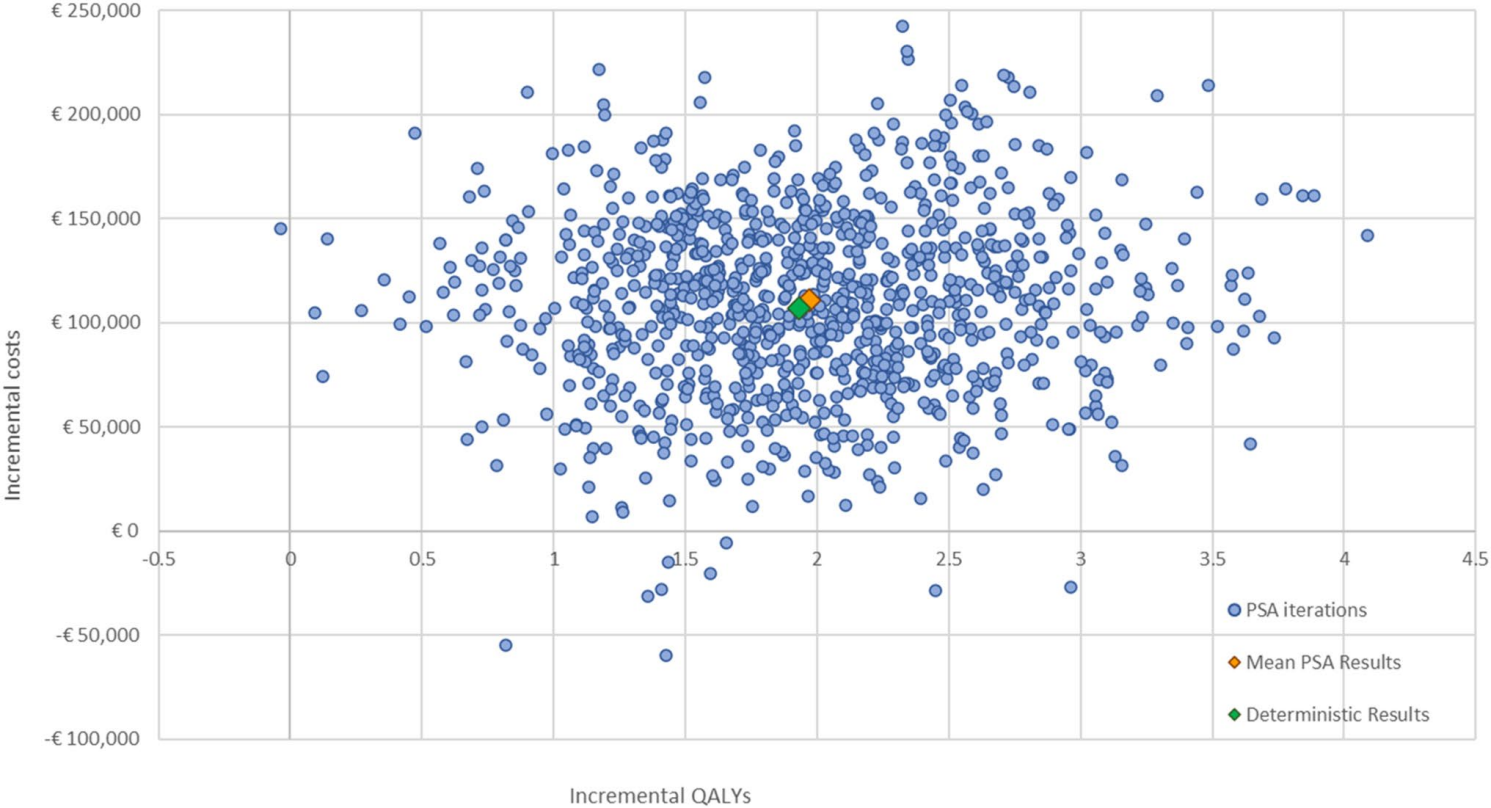
Cost-effectiveness plane of T-DXd versus T-DM1. *PSA* probabilistic sensitivity analysis, *QALY* quality-adjusted life year

Figure 4 shows the cost-effectiveness plane of T-DXd versus T-DM1, where the incremental health outcomes (in QALYs) of T-DXd are plotted against the incremental costs of T-DXd for 1000 iterations. Most iterations (approximately 99%) indicate that T-DXd has higher costs and more QALYs gained compared to T-DM1.

In the absence of a clear willingness-to-pay (WTP) threshold in Finland, the results of the Fimea assessment of T-DXd as a third-line treatment in HER2-positive mBC were used as a surrogate WTP threshold that are likely to be accepted by decision makers. Two surrogate thresholds were identified: €72,000 per QALY and €139,000 per QALY [10]. The cost-effectiveness acceptability curve showed that T-DXd versus T-DM1 has a likelihood of 68.40% and 95.40% of being cost-effective versus the surrogate WTP thresholds of €72,000 per QALY and €139,000 per QALY, respectively (Fig. 5—Supplementary Information 1).

#### Scenario analysis

A scenario using rates of subsequent treatment from Cortés et al.—29.9% for the T-DXd arm and 62.4% for the T-DM1 arm—resulted in an ICER of €55,135 per QALY [9]. Results from scenarios exploring utility values for the progression-free and post-progression health states did not differ substantially from the base case ICER. The greatest effect was seen when applying treatment-specific health state utilities from DB-03 to both health states (€64,136 per QALY), followed by applying utilities derived from Lloyd with DB-03 patient characteristics to both health states (€58,012 per QALY), and applying non-treatment-specific health state utilities from DB-03 to both health states (€55,452 per QALY). Extrapolating the DB-03 OS data using the direct extrapolation resulted in an ICER of €68,588 per QALY. Results of all scenario analyses are shown in Table 9—Supplementary Information 1.

## Discussion

T-DXd was recently recommended as a second-line treatment for HER2-positive mBC in the ESMO clinical guidelines [7]. This recommendation was based on the improved survival outcomes for T-DXd compared with T-DM1 as second-line treatment for HER2-positive mBC demonstrated in the DB-03 clinical trial [9]. This is the first study to evaluate the cost-effectiveness of T-DXd in this patient population. We used DB-03 trial results to conduct a cost-effectiveness analysis comparing T-DXd with T-DM1 for the second-line treatment after trastuzumab and a taxane of HER2-positive uBC and/or mBC from the Finnish payer’s perspective. The results show that, using prices from Kela's medicinal products, T-DXd provides a QALY gain of 1.93 versus T-DM1 and increases lifetime costs by €106,800, resulting in an ICER of €55,360 per QALY. The PSA results support the base case, yielding a mean probabilistic ICER of €56,084 per QALY. Previous related Fimea assessments have reported higher ICERs of €72,000 per QALY and €139,000 per QALY [10]. Using these ICERs as surrogate WTP thresholds indicates that the results for T-DXd versus T-DM1 can be considered cost-effective.

The OWSA showed that health state utilities were among the most influential parameters. The base case uses DB-03 treatment-specific utility values for the progression-free health state to account for the impact of response rate on the quality of life. A scenario with non-treatment-specific utility values from DB-03 yielded an ICER of €55,452 per QALY, showing that the base case assumption does not have a major influence on the ICER.

The partitioned survival approach allowed for direct estimates of health state membership to be derived from the survival curves. Though PartSA is commonly used for assessing cost-effectiveness for new interventions in metastatic or advanced cancers in the NICE Technology Appraisal Programme—generally considered to represent the highest standards for cost-effectiveness analyses—a limitation of these models is the uncertainty around the extrapolated survival curves, particularly where data is immature. Survival data from DB-03 for TTD and PFS was mature and could be directly extrapolated using a standard parametric model; however, because the DB-03 OS event rate was low when our analysis was conducted, an alternative approach was used in an attempt to reduce the uncertainty for this outcome. This approach (outlined in detail in the method section) leveraged a broader wealth of available clinical information by combining data from different relevant sources; however, it is not without its limitations.

The alternative approach considers both proportional hazard and accelerated failure time (AFT) models for the extrapolation of long-term EMILIA OS data. The extrapolated EMILIA data and the DB-03 T-DM1 data were then each used to calculate hazard rate functions. These functions were combined into one hazard rate function from which the T-DM1 OS curve could be calculated. Afterwards, the approach applies a HR to the T-DM1 OS curve and used DB-03 T-DXd data to determine the T-DXd OS curve. A limitation of AFT models is that a HR cannot be applied to it directly since AFT models do not have a constant hazard rate. However, the HR was not applied directly to the extrapolated long-term EMILIA OS data, rather it was applied to the OS curve derived from the combined hazard rate function. Furthermore, AFT models, especially the generalized gamma, provided the best statistical fit to the long-term OS data. The generalized gamma was also shown to provide the most clinically plausible survival estimates in the clinical plausibility analysis (Supplementary Information 2). Therefore, the generalized gamma was chosen in the base case. The HR used in this approach was found to have substantial influence on the ICER in the OWSA. This HR directly influences the OS curve for T-DXd, thereby impacting health state membership and the ICER. Since the DB-03 OS event rate was low, this HR introduces uncertainty into the model. A scenario where the DB-03 OS data was directly extrapolated resulted in an ICER of €68,570 per QALY. However, direct extrapolation of the OS curves could result in uncertainty given the amount of censored OS observations at the time of conducting this study. Furthermore, the use of external data for long-term model extrapolation has shown to be of higher predictive power [25, 26]. Noteworthy, using both extrapolation methods, T-DXd remains cost-effective using the surrogate WTP thresholds.

The assumptions and choices made for modelling subsequent treatment also present limitations for this analysis. First, the assumption that all patients are administered subsequent treatment until death possibly overestimates the actual subsequent treatment costs. Secondly, T-DXd has been approved for the third-line treatment of HER2-positive uBC and/or mBC after T-DM1, but no information is available on the effectiveness of T-DM1 after second-line T-DXd treatment. Thus, there is uncertainty as to whether the modelled subsequent treatment costs will be reflective of clinical practice.

A final limitation is the inclusion of Grade 3 or higher AEs with an incidence of ≥ 5% in one or both arms of DB-03. This threshold could potentially exclude less common but still relevant AEs, and we included AEs of special interest in the model to account for this. There were also assumptions made for the costs of treating AEs—specifically, it was assumed that AE-related costs were only incurred in the first cycle of the model, concurrent with treatment initiation. We did not run a scenario analysis for this input, so the impact on the ICER is unknown.

It is a strength of this model that it is primarily based on clinical trial data from DB-03, with additional data from EMILIA. However, an update of this model using real-world evidence or long-term trial data could reduce uncertainties in current model predictions. Nonetheless, the alternative approach to direct extrapolation used in this study could be a useful tool and presents an opportunity for further research into cost-effectiveness analysis where not all survival data is mature.

## Conclusion

This cost-effectiveness analysis showed that, as a second-line treatment for HER2-positive uBC and/or mBC, T-DXd accrues an additional 1.93 QALYs and €106,800 per patient over a lifetime compared with the previous standard of care, T-DM1. This results in an ICER of €55,360 per QALY. Based on surrogate WTP thresholds of €72,000 per QALY and €139,000 per QALY, T-DXd is cost-effective.

### Supplementary Information

Below is the link to the electronic supplementary material.

Supplementary file1 (DOCX 576 KB)

Supplementary file2 (DOCX 198 KB)

## Data Availability

Most data are included in the manuscript and its supplementary files. The analyses were largely conducted based on publicly available information which is presented and referenced in the article and supplementary files. Some of the data generated during and/or analysed during the current study are not publicly available, but are available from the corresponding author on request.
