# Supplementary material for: Cost-effectiveness model of trastuzumab deruxtecan as second-line treatment in HER2-positive unresectable and/or metastatic breast cancer in Finland
Source: Eur J Health Econ. 2023 Jul 24;25(4):689–99. doi: 10.1007/s10198-023-01617-3 (PMC11136791; doi:10.1007/s10198-023-01617-3)
Supplement: Supplementary file 1 — Supplementary file1 (DOCX 576 KB) [file 10198_2023_1617_MOESM1_ESM.docx]

**Cost-effectiveness model of trastuzumab deruxtecan as second-line treatment in HER2-positive unresectable and/or metastatic breast cancer in Finland — Supplementary information 1**

**List of distribution parameters**

Table 1 List of distribution parameters

| **Distribution** | **Parameter** |
| --- | --- |
| Exponential | Rate |
| Weibull | Scale |
|  | Shape |
| Gompertz | Scale |
|  | Shape |
| Log-Logistic | Scale |
|  | Shape |
| Log normal | Meanlog |
|  | Sdlog |
| Generalized gamma | Mu |
|  | Sigma |
|  | Q |
| Gamma | Scale |
|  | Shape |

**PFS T-DXd**

Table 2 Goodness of fit for T-DXd to the DB-03 PFS data according to AIC & BIC, unpublished DB-03 clinical study report

| Distribution | AIC | BIC |
| --- | --- | --- |
| Exponential | 811.150 | 814.720 |
| Weibull | 804.180 | 811.310 |
| Gompertz | 809.640 | 816.770 |
| Log-Logistic | 802.000 | 809.130 |
| **Log normal** | **800.830** | **807.960** |
| Generalized gamma | 802.770 | 813.460 |
| Gamma | 811.150 | 814.720 |

Abbreviations: AIC, Akaike Information Criterion; BIC, Bayesian Information Criterion; DB-03, DESTINY-BREAST03; PFS, progression-free survival; T-DXd, fam-trastuzumab deruxtecan-nxki.


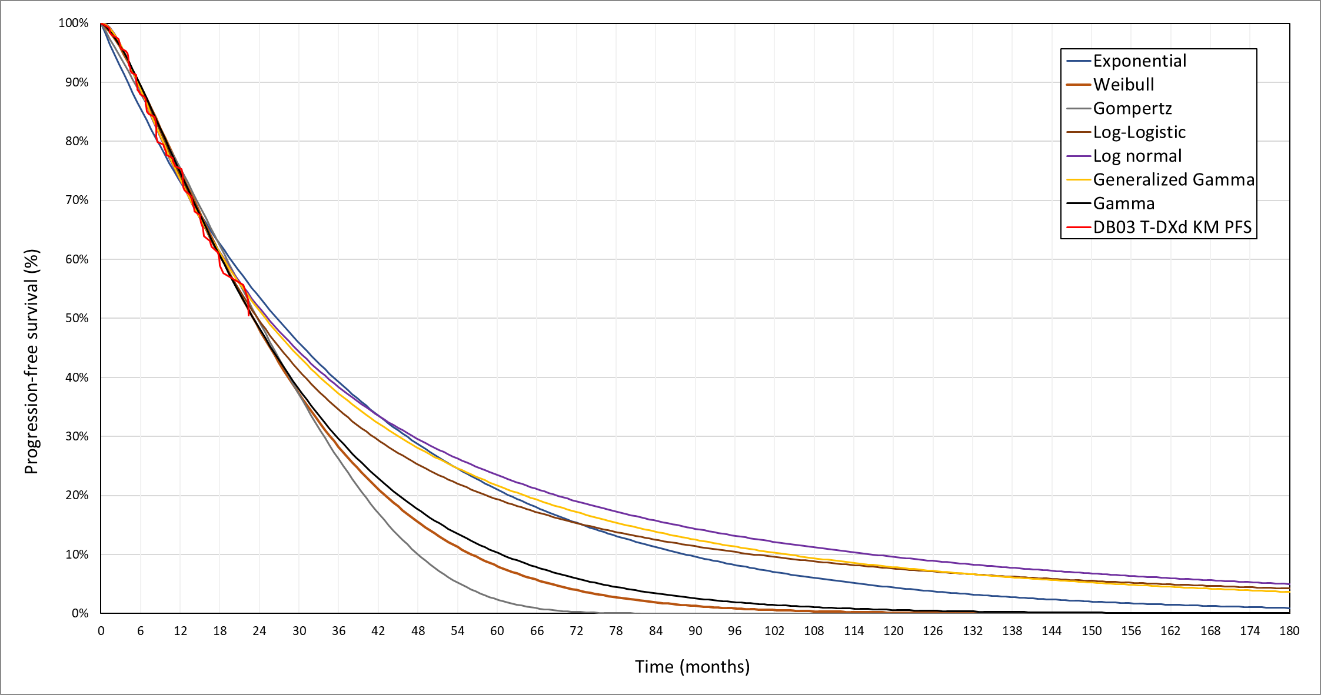


**Fig. 1** Standard parametric survival extrapolations of T-DXd PFS with KM curve of DB-03 T-DXd shown

Abbreviations: DB-03, DESTINY-BREAST03; KM, Kaplan-Meier; PFS, progression-free survival; T-DXd, fam-trastuzumab deruxtecan-nxki.

Table 3 Median PFS of T-DXd extrapolation compared to observed median PFS by investigator review in DB-03 for T-DXd, unpublished DB-03 clinical study report

| Distribution | Median PFS |
| --- | --- |
| **Observed median PFS in DB-03** | **25.1 months** |
| Exponential | 26.3 months |
| Weibull | 22.8 months |
| Gompertz | 23.5 months |
| Log-Logistic | 23.5 months |
| **Log normal** | **24.9 months** |
| Generalized gamma | 24.2 months |
| Gamma | 22.8 months |

Abbreviations: DB-03, DESTINY-BREAST03; PFS, progression-free survival; T-DXd, fam-trastuzumab deruxtecan-nxki.

**PFS T-DM1**

Table 4 Goodness of fit for T-DM1 to the DB-03 PFS data according to the AIC & BIC, unpublished DB-03 clinical study report

| Distribution | AIC | BIC |
| --- | --- | --- |
| Exponential | 1091.100 | 1094.670 |
| Weibull | 1093.030 | 1100.170 |
| Gompertz | 1081.180 | 1088.330 |
| Log-Logistic | 1067.400 | 1074.540 |
| Log normal | 1058.420 | 1065.560 |
| **Generalized gamma** | **1045.190** | **1055.910** |
| Gamma | 1092.600 | 1099.740 |

Abbreviations: AIC, Akaike Information Criterion; BIC, Bayesian Information Criterion; DB-03, DESTINY-BREAST03; PFS, progression-free survival; T-DM1, ado-trastuzumab emtansine.


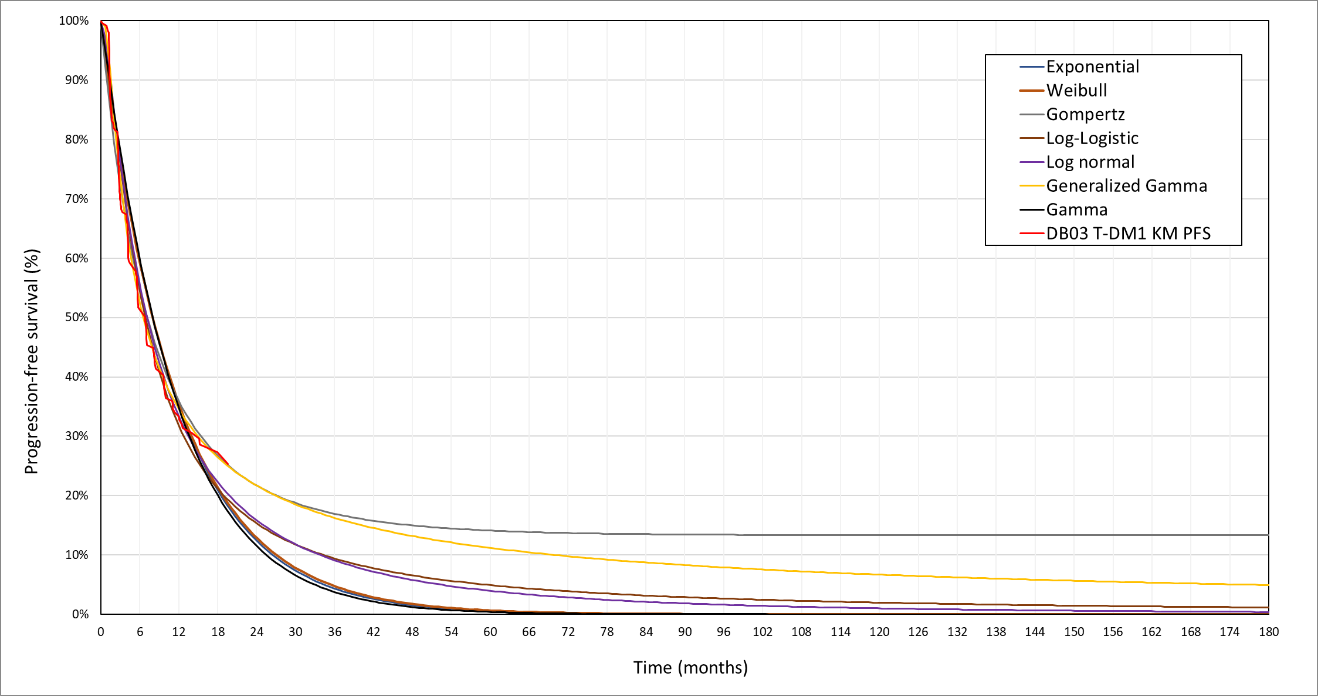


**Fig. 2** Standard parametric survival extrapolations of T-DM1 PFS with KM curve of DB-03 T-DM1 shown
Abbreviations: DB-03, DESTINY-BREAST03; KM, Kaplan-Meier; PFS, progression-free survival; T-DM1, ado-trastuzumab emtansine.

Table 5 Median PFS of T-DM1 extrapolations compared to observed median PFS by BICR for T-DM1, unpublished DB-03 clinical study report

| Distribution | Median PFS |
| --- | --- |
| **Observed median PFS in DB-03** | **6.8 months** |
| Exponential | 7.6 months |
| Weibull | 7.6 months |
| **Gompertz** | **6.9 months** |
| Log-Logistic | 6.2 months |
| **Log normal** | **6.9 months** |
| Generalized gamma | 6.2 months |
| Gamma | 7.6 months |

Abbreviations: BICR, blinded independent central review; DB-03, DESTINY-BREAST03; PFS, progression-free survival; T-DM1, ado-trastuzumab emtansine.

**OS T-DXd & T-DM1**

Table 6 Goodness of fit for T-DM1 to the long-term EMILIA data according to the AIC & BIC, unpublished DB-03 clinical study report

| Distribution | AIC | BIC |
| --- | --- | --- |
| Exponential | 2926.343 | 2930.548 |
| Weibull | 2879.742 | 2888.151 |
| Gompertz | 2913.151 | 2921.56 |
| Log-Logistic | 2852.867 | 2861.276 |
| Log normal | 2843.331 | **2851.74** |
| **Generalized gamma** | **2841.971** | 2854.585 |
| Gamma | 2867.304 | 2875.713 |

Abbreviations: AIC, Akaike Information Criterion; BIC, Bayesian Information Criterion; T-DM1, ado-trastuzumab emtansine.


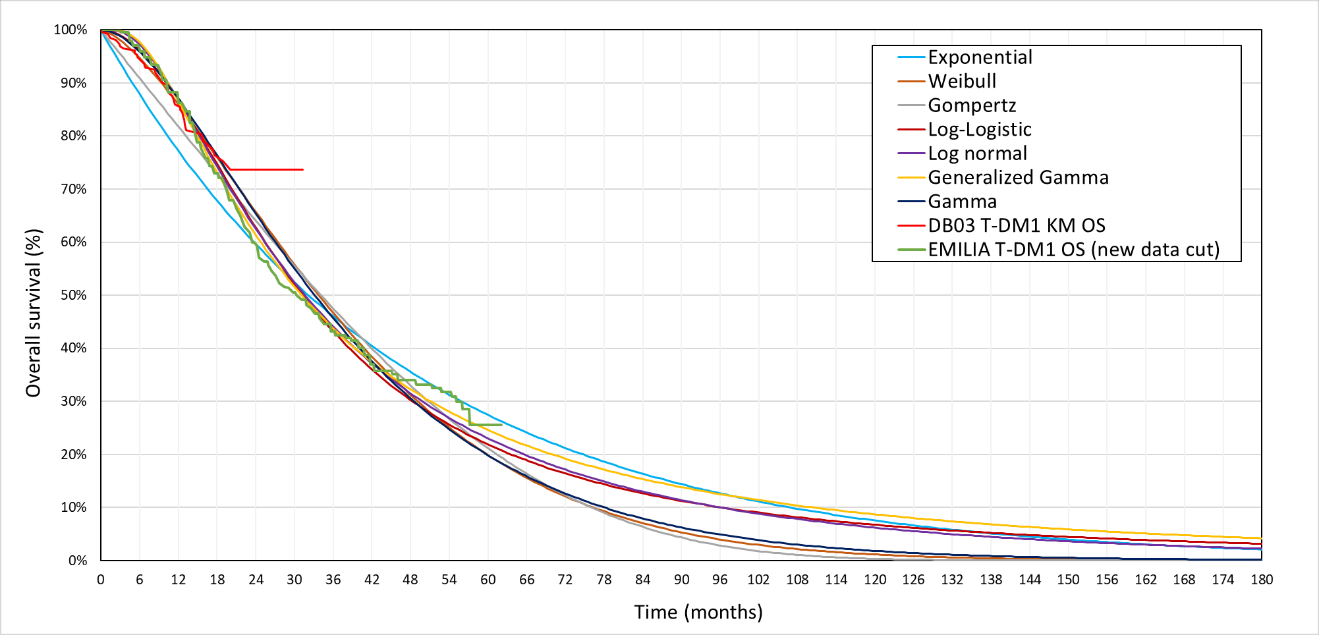


**Fig. 3** Standard parametric survival extrapolations of long-term EMILIA T-DM1 OS with reconstructed KM plot of EMILIA and the KM curve of DB-03 T-DM1 shown
Abbreviations: DB-03, DESTINY-BREAST 03; KM, Kaplan-Meier; OS, overall survival; T-DM1, ado-trastuzumab emtansine.


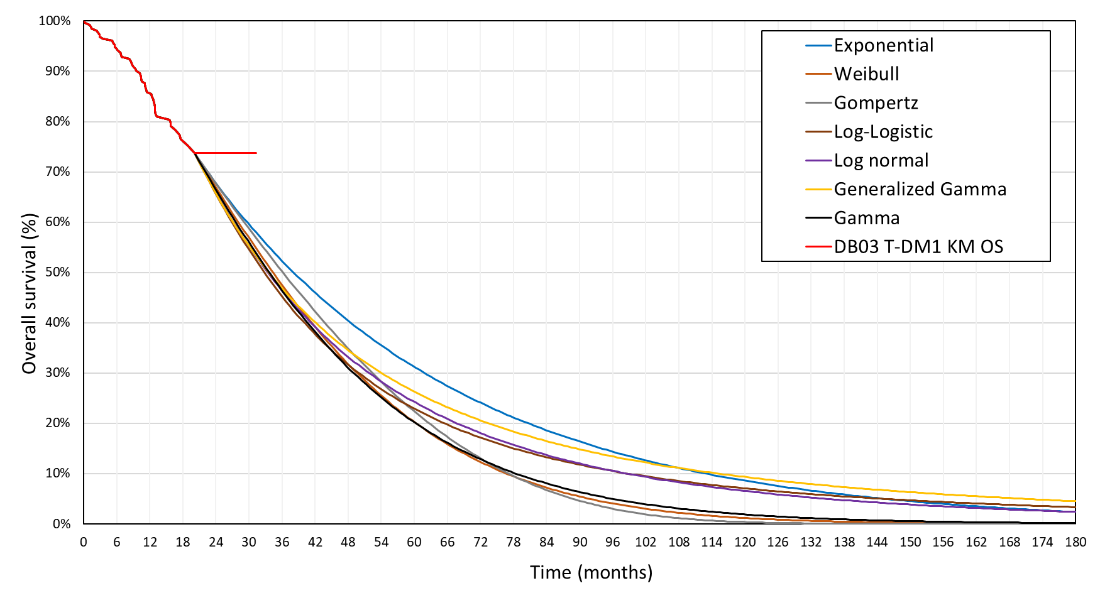


**Fig. 4** Standard parametric survival extrapolations of T-DM1 OS data and resulting survival curves of T-DM1 based on KM + Tail method
Abbreviations: DB-03, DESTINY-BREAST03; KM, Kaplan-Meier; OS, overall survival; T-DM1, ado-trastuzumab emtansine.

**Utility calculations**

Equation 1: Lloyd algorithm for calculating utility values. [1]

$$Utility value= \frac{exp(sum of coefficients)}{1+exp(sum of coefficients)}$$

To calculate utility values for the cost-effectiveness model, the intercept, the age coefficient, and the disease progression coefficient were used. The mixed model analysis performed by Lloyd et al. (2006) resulted in the following coefficients: the intercept is 0.008871, the coefficient for age is 0.0239 (to be multiplied by age), and the coefficient for disease progression is -1.1477 [2]. Adding these coefficients and the mean age of DB03—i.e., 54.4—into Equation S1 results in Equation S2:

Equation 2: Calculated PP utility value

$$Utility value PP= \frac{exp(0.008871+0.239\times54.4 + -1.1477)}{1+exp(0.008871+0.239\times54.4 + -1.1477)}=0.5403$$

Table 7 Subsequent treatment regimen costs and proportion of patients receiving each regimen

| **Subsequent treatment regimen** | **Cost per pack** | **Proportion of patients receiving treatment—T-DXd arm** | **Proportion of patients receiving treatment—T-DM1 arm** | **Source** |
| --- | --- | --- | --- | --- |
| Trastuzumab/Lapatinib + chemo | €20,342 | 50% | 10% | [2] |
| T-DXd | €145,964 | 0% | 70% | [3] |
| T-DM1 | €71,729 | 10% | 0% | [2] |
| Tucatinib^a^ + capecitabine + trastuzumab | €93,015 | 40% | 20% | Calculated*  [3] |

Abbreviations: DB-03, DESTINY-Breast03; HER2, human epidermal growth factor receptor 2; IHS, Information Handling Services; T-DM1, ado-trastuzumab emtansine T-DXd, fam-trastuzumab deruxtecan-nxki.

Notes: 7.6 (months PFS in HER2Climb)*4.35(weeks per months) /3 (cycle length in weeks)*7645.96 (cost per pack of 84 150mg tables) /1.1 (remove VAT)*0.885 (RDI)+€25,225 (Trastuzumab + cytotoxic agent costs)

**Results**

Table 8 Probabilistic results, disaggregated costs, health gains, and time-to-event outcomes of T-DXd versus T-DM1

| Probabilistic results | |
| --- | --- |
| ICER (Cost/QALY) | €56,084 |
| ICER (Cost/LY) | €42,656 |
| Incremental costs | €110,578 |
| Incremental QALYs | 1.97 |
| Incremental LYs | 2.59 |

Abbreviations: ICER, incremental cost-effectiveness ratio; LY, life year; QALY, quality-adjusted life year;

**
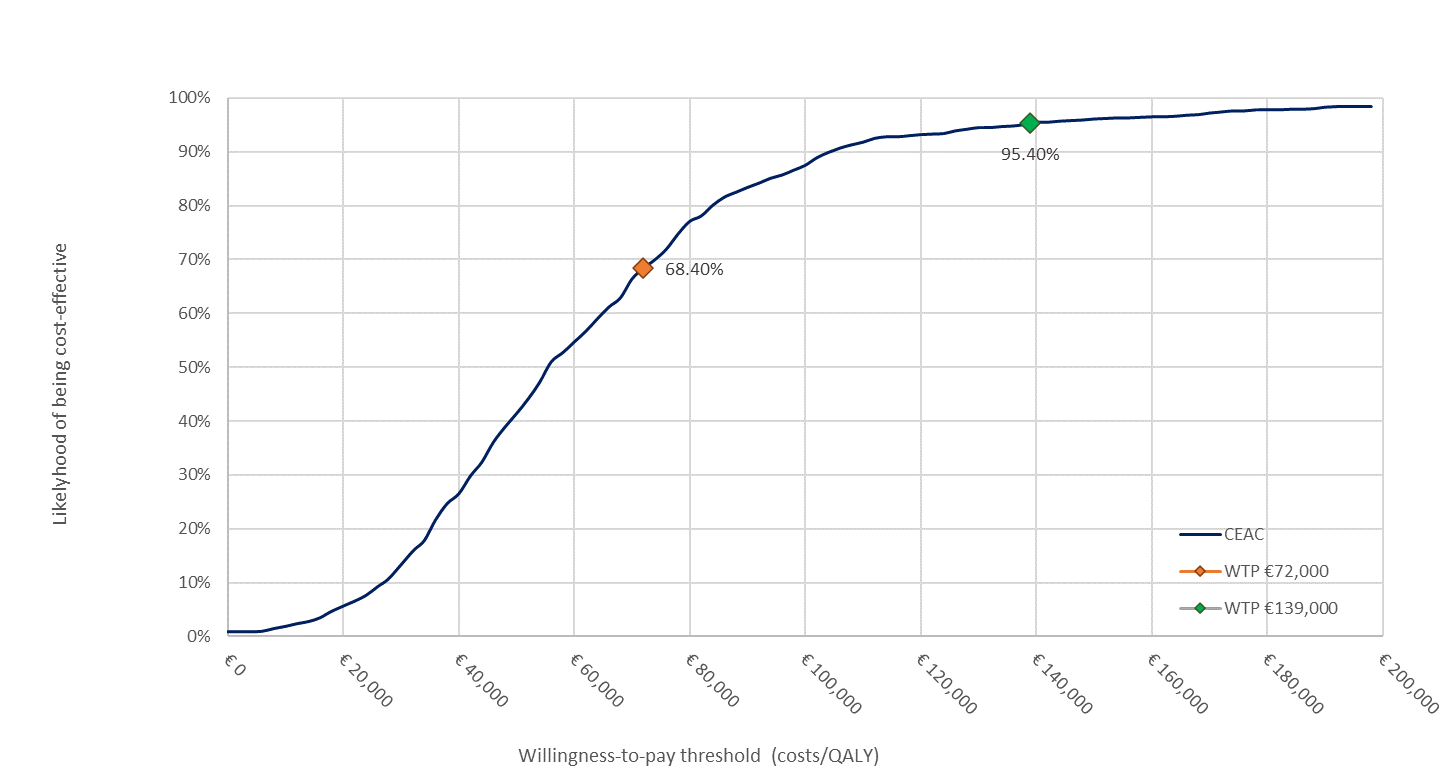
**

**Fig. 5** Cost-effectiveness acceptability curve of T-DXd

Table 9 Results of the scenario analyses

| **#** | **Scenario** | **ΔCosts** | **ΔQALYs** | **ICER** |
| --- | --- | --- | --- | --- |
|  | **Base case** | **€106,800** | **1.93** | **€55,360** |
| 1 | Discount rates - C: 0%, H: 0% | €127,789 | 2.69 | €47,559 |
| 2 | Discount rates - C: 6%, H: 6% | €91,204 | 1.47 | €61,921 |
| 3 | Time horizon - 5 years | €69,654 | 0.66 | €105,770 |
| 4 | Time horizon - 10 years | €93,836 | 1.18 | €79,787 |
| 5 | Time horizon - 30 years | €106,143 | 1.87 | €56,791 |
| 6 | Half-cycle correction - No | €107,399 | 1.93 | €55,715 |
| 7 | Background mortality - No | €108,222 | 2.23 | €48,554 |
| 8 | Vial Sharing - 100% | €94,869 | 1.93 | €49,175 |
| 9 | Vial Sharing - 0% | €118,731 | 1.93 | €61,544 |
| 10 | Pts receiving subs txt T-DXd: 29.9%, T-DM1: 62.4% | €106,367 | 1.93 | €55,135 |
| 11 | Health state utilities: DB-03 | €106,800 | 1.67 | €64,136 |
| 12 | Health state utilities: DB-03 + Lloyd | €106,800 | 1.84 | €58,012 |
| 13 | Health state utilities: DB-03 - overall | €106,800 | 1.93 | €55,452 |
| 14 | Direct extrapolation OS; log-logistic | €105,130 | 1.53 | €68,588 |

Abbreviations: DB-03, DESTINY-Breast 03; OS, overall survival; PFS, progression-free survival; T-DM1, ado-trastuzumab emtansine; T-DXd, fam-trastuzumab deruxtecan-nxki; TTD, time to treatment discontinuation.

**References**

1. Lloyd, A., Nafees, B., Narewska, J., Dewilde, S., Watkins, J.: Health state utilities for metastatic breast cancer. Br J Cancer. 95, 683–690 (2006). https://doi.org/10.1038/SJ.BJC.6603326

2. Härkönen, U., Itkonen, L., Kiviniemi, V., Oravilahti, T.: Trastutsumabiemtansiini HER2-positiivisen rintasyövän hoidossa. In: Fimea kehittää, arvioi ja informoi -julkaisusarja. Fimea (Finnish Medicines Agency), Helsinki (2015)

3. Kotajärvi, J., Hyvärinen, A., Wikman, E., Kiviniemi, V.: Trastutsumabi-derukstekaani levinneen HER2-positiivisen rintasyövän hoidossa: Uusien sairaalalääkkeiden arviointi. In: Fimea kehittää, arvioi ja informoi . Fimea (Finnish Medicines Agency), Kuopio (2021)
