# Supplementary material for: Cost-effectiveness model of trastuzumab deruxtecan as second-line treatment in HER2-positive unresectable and/or metastatic breast cancer in Finland
Source: Eur J Health Econ. 2023 Jul 24;25(4):689–99. doi: 10.1007/s10198-023-01617-3 (PMC11136791; doi:10.1007/s10198-023-01617-3)
Supplement: Supplementary file 2 — Supplementary file2 (DOCX 198 KB) [file 10198_2023_1617_MOESM2_ESM.docx]

**Cost-effectiveness model of trastuzumab deruxtecan as second-line treatment in HER2-positive unresectable and/or metastatic breast cancer in Finland— Supplementary information 2**

**Validation of the OS method**

The first method to validate the use of EMILIA data compared the T-DM1 KM plots from EMILIA, the unpublished DB-03 clinical study report, and KATE2 [1, 2]. KATE2 data, although immature, were included to account for the difference in previous pertuzumab use between EMILIA and DB-03. Patients in the KATE2 trial had a comparable percentage of prior pertuzumab use to patients in DB-03 (48% and 61%, respectively) [1]. The comparison between the T-DM1 KM plots from EMILIA, DB-03, and KATE2 showed a clear overlap (Figure 1).

The subgroup analysis for patients treated with prior pertuzumab in the unpublished DB-03 clinical study report showed comparable HRs for subjects with (HR = 0.30 [95%CI: 0.22–0.43]) and without (HR = 0.30 [95%CI: 0.19–0.47]) prior pertuzumab treatment.

In conclusion, based on these validations described above, the EMILIA T-DM1 arm was chosen to reflect anticipated long-term outcomes for the DB-03 T-DM1 arm.
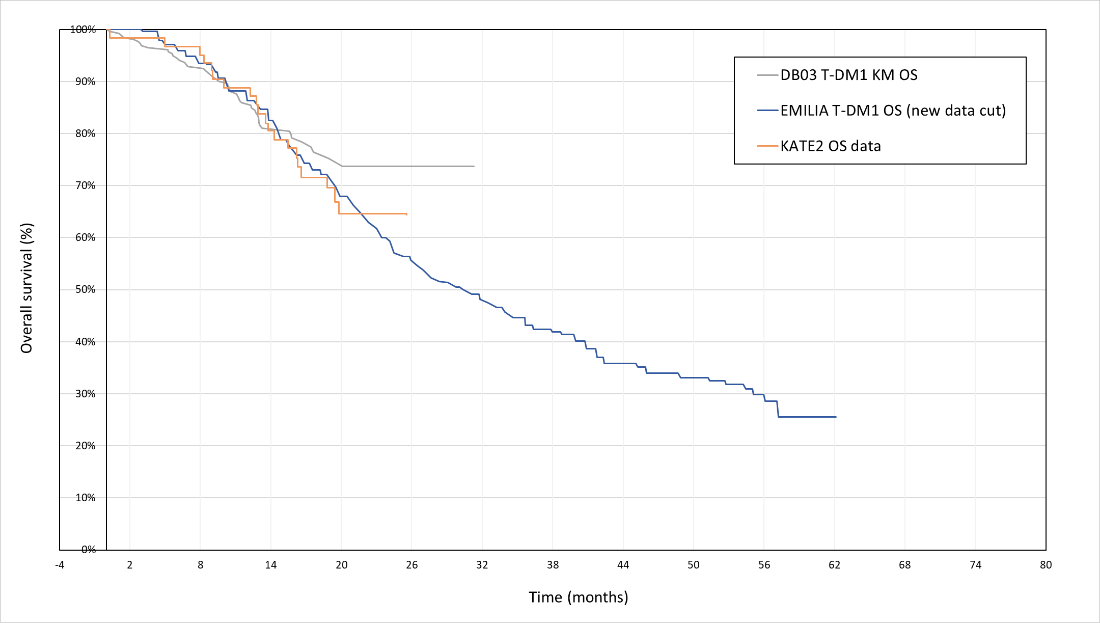

**Fig. 1** KM estimates for OS of the T-DM1 arms from EMILIA, KATE2 and DB-03. Abbreviations: DB-03, DESTINY-BREAST03; KM, Kaplan-Meier; OS, overall survival; T-DM1, ado-trastuzumab emtansine.

**Additional analysis of clinical plausibility — OS parametric distributions**

Hazard plots were created for the following parameters: hazard rates for OS of T-DXd and T-DM1 as second-line treatments (using data from DB-03); OS of T-DXd as third-line treatment (using digitized data from DESTINY-Breast01 [3]); OS of T-DM1 as second-line or later treatment (using digitized data from EMILIA [2]); and pertuzumab, trastuzumab, and docetaxel as first-line treatment (using digitized data from CLEOPATRA [4]; Figure 2). The hazard plots showed a similar pattern in hazard rates for T-DXd as third-line treatment and T-DM1 as second-line treatment and a similar pattern for T-DXd as second-line treatment and pertuzumab, trastuzumab, and docetaxel as first-line treatment. Figure 3 shows that the generalized gamma distribution created an OS-curve for T-DXd that followed the trend observed in the hazard plots.


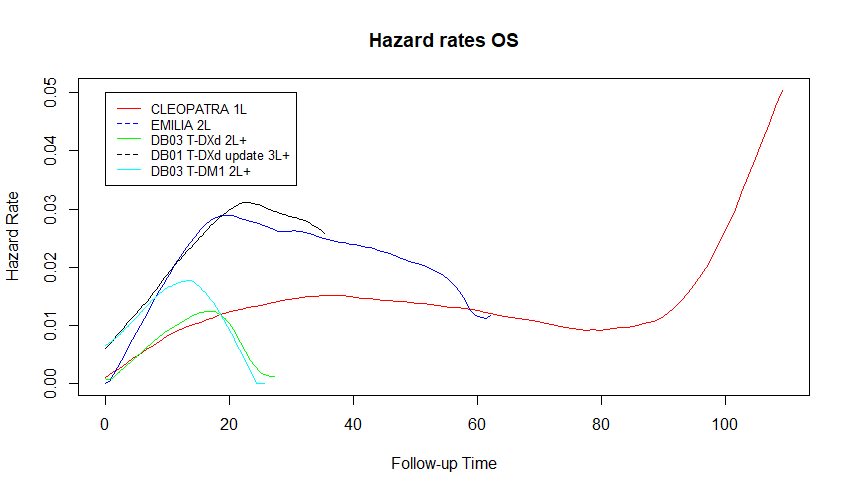


**Fig. 2** Hazard plot of targeted HER2+ agents comparting T-DXd to standard therapies in an earlier line
Note: The drop at approximately 20 months in DB-03 data is attributable to the immaturity of the data. Abbreviations: 1L, first line; 2L, second line; 3L, third line; DB-03, DESTINY-BREAST03; KM, Kaplan-Meier; OS, overall survival; T-DM1, ado-tratuzumab emtansine; T-DXd, fam-trastuzumab deruxtecan-nxki.


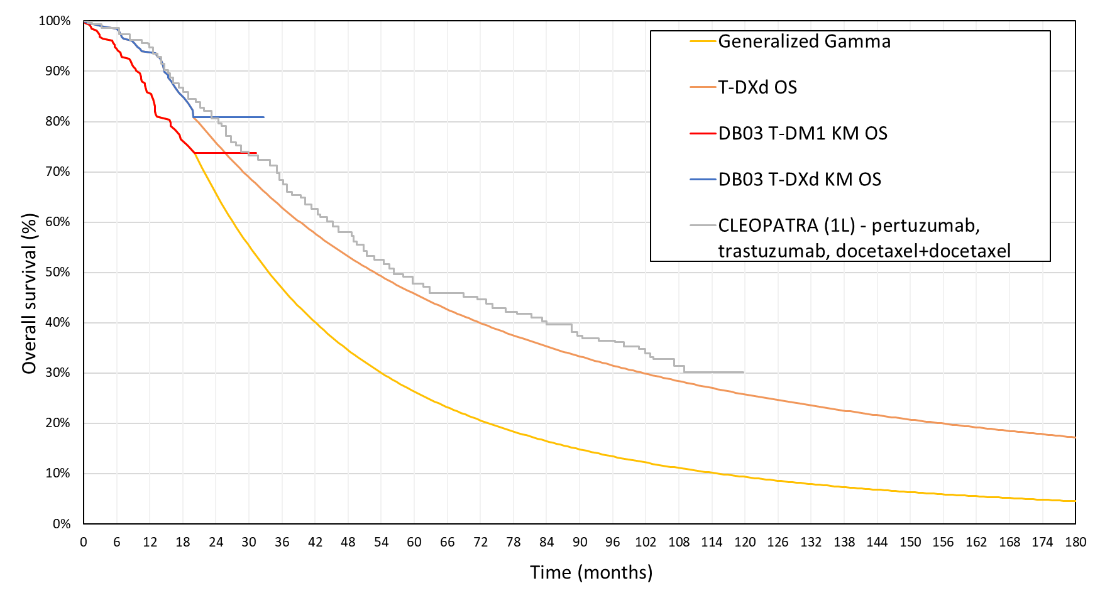


**Fig. 3** Generalized gamma extrapolation of T-DM1 OS data and resulting survival curve for T-DXd compared to digitized OS KM-curve for pertuzumab, trastuzumab, and docetaxel as first-line treatment from CLEOPATRA.

Abbreviations: DB-03, DESTINY-BREAST03; KM, Kaplan-Meier; OS, overall survival; T-DM1, ado-tratuzumab emtansine; T-DXd, fam-trastuzumab deruxtecan-nxki.

**References**

1. Emens, L.A., Esteva, F.J., Beresford, M., Saura, C., de Laurentiis, M., Kim, S.B., Im, S.A., Wang, Y., Salgado, R., Mani, A., Shah, J., Lambertini, C., Liu, H., de Haas, S.L., Patre, M., Loi, S.: Trastuzumab emtansine plus atezolizumab versus trastuzumab emtansine plus placebo in previously treated, HER2-positive advanced breast cancer (KATE2): a phase 2, multicentre, randomised, double-blind trial. Lancet Oncol. 21, 1283–1295 (2020). https://doi.org/10.1016/S1470-2045(20)30465-4

2. Diéras, V., Miles, D., Verma, S., Pegram, M., Welslau, M., Baselga, J., Krop, I.E., Blackwell, K., Hoersch, S., Xu, J., Green, M., Gianni, L.: Trastuzumab emtansine versus capecitabine plus lapatinib in patients with previously treated HER2-positive advanced breast cancer (EMILIA): a descriptive analysis of final overall survival results from a randomised, open-label, phase 3 trial. Lancet Oncol. 18, 732–742 (2017). https://doi.org/10.1016/S1470-2045(17)30312-1

3. Modi, S., Saura, C., Yamashita, T., Park, Y.H., Kim, S.B., Tamura, K., Andre, F., Iwata, H., Ito, Y., Tsurutani, J., Sohn, J., Denduluri, N., Perrin, C., Aogi, K., Tokunaga, E., Im, S.A., Lee, K.S., Hurvitz, S.A., Cortes, J., Lee, C., Chen, S., Zhang, L., Shahidi, J., Yver, A., Krop, I.: Trastuzumab deruxtecan in previously treated HER2-positive breast cancer. New England Journal of Medicine. 382, 610–621 (2020). https://doi.org/10.1056/NEJMoa1914510

4. Swain, S.M., Miles, D., Kim, S.B., Im, Y.H., Im, S.A., Semiglazov, V., Ciruelos, E., Schneeweiss, A., Loi, S., Monturus, E., Clark, E., Knott, A., Restuccia, E., Benyunes, M.C., Cortés, J., Agajanian, R., Ahmad, R., Aktas, B., Alencar, V.H., Amadori, D., Andrade, J., André Franke, F., Angiolini, C., Aogi, K., Armor, J., Arpornwirat, W., Assersohn, L., Audeh, W., Aulitzky, W., Azevedo, S., Bartoli, M.A., Batista Lopez, N., Bianconi, M., Biganzoli, L., Birhiray, R., Bitina, M., Blachy, R., Blackwell, K., Blanchard, R., Blanchet, P., Boiangiu, I., Bower, B., Brezden-Masley, C., Brufsky, A., Budde, L., Caguioa, P., Calvo, L., Campone, M., Carroll, R.R., Castro, H., Chan, V., Charu, V., Cinieri, S., Clemens, M., Conejo, E.A., Côrtes, E., Coudert, B., Cronemberger, E., Cubero, D., Dakhil, S., Daniel, B., Davidson, N., de Fatima Gaui, M., de La Cruz, S., del Pilar, M., Delgado, G., Ellerton, J.A., Estuardo, C., Fehrenbacher, L., Ferrero, J.M., Flynn, P.J., Foszczynska-Kloda, M., Franco, S., Fujii, H., Gallagher, C., Gamucci, T., Giacomi, N., Gil I Gil, M., Gonzalez Martin, A., Gorbunova, V., Gotovkin, E., Green, N., Grincuka, E., Grischke, E.M., Hansen, V., Hargis, J., Hauschild, M., Hegg, R., Hendricks, C., Hermann, R., Hoff, P., Horiguchi, J., Hornedo Muguiro, J., Iacobelli, S., Inoue, K., Ismael, G., Itoh, Y., Iwata, D.H., Jendiroba, D., Jochim, R., Jones, A., Just, M., Kallab, A., Karwal, M., Kashiwaba, M., Kato, G., Kaufman, P.A., Kellokumpu-Lehtinen, P., Kirsch, A., Kiselev, I., Klein, P., Kohno, N., Kopp, M., Kostovska-Maneva, L., Kotliar, M., Kudaba, I., Kümmel, S., Kuroi, K., Lacava, J., Latini, L., Lee, S.C., Lichinitser, M., Lobo, C., Maintz, C., Maneecahvakajorn, J., Marmé, A., Martinez, G., Masuda, N., Matwiejuk, M., Merculov, V., Michaelson, R., Miguel, L., Monroy, H., Montemurro, F., Morales, S., Moura, R., Mueller, V., Mulatero, C., Nakagami, K., Nakayama, T., Neidhart, J., Nguyen, A., Nishimura, R., Ogata, H., O’reilly, S., O’rourke, T., Otero Reye, D., Ouyang, X., Patel, R., Patel, T., Pedrini, J.L., Pereira, R., Perez, A., Peterson, C., Pienkowski, T., Pinczowski, H., Polikoff, J., Polkowski, W., Price, P.E., Prill, S., Priou, F., Purkalne, G., Pyrhoenen, S., Quackenbush, R., Rai, Y., Ribelles, N., Ro, J., Robinson, A., Robles, R., Rodriguez, G., Roman, L., Saji, S., Sanchez-Rovira, P., Sato, N., Schmidt, M., Schumacher, C., Senecal, F., Sharma, P., Shen, Z., Shirinkin, V., Simoncini, E., Sirisinha, T., Smith, R., Sohn, J.H., Soldic, Z., Soria, T., Spicer, D., Srimuninnimit, V., Sriuranpong, V., Staroslawska, E., Stefanovski, P., Sunpaweravong, P., Taguchi, J., Takeda, K., Tellez-Trevilla, G., Thomas, R., Thomssen, C., Toache, Z., Tokuda, Y., Tomczak, P., Tosello, C., Tsugawa, K., Tudtud, D., Ueno, T., van Eyll, B., Varela, M., Vasev, N., Vrbanec, D., Wang, X., Wang, L., Watanabe, J., Waterhouse, D., Wesenberg, B., Wheatley, D., Wong, Z.W., Yadav, S., Yardley, D., Yau, T.K., Yeo, W., Ying, C., Youn Oh, D.: Pertuzumab, trastuzumab, and docetaxel for HER2-positive metastatic breast cancer (CLEOPATRA): end-of-study results from a double-blind, randomised, placebo-controlled, phase 3 study. Lancet Oncol. 21, 519–530 (2020). https://doi.org/10.1016/S1470-2045(19)30863-0
